# Implantation Metastasis of Laryngeal Neuroendocrine Carcinoma at Tracheotomy Site

**DOI:** 10.1002/ccr3.72511

**Published:** 2026-04-12

**Authors:** Xiaolong Hao, Rui Hou, Shuxin Wen, Yuhao Zhang, Tian Wang, Xiaolin Ji, Yueqin Wang

**Affiliations:** ^1^ Department of Otolaryngology Head and Neck Surgery Shanxi Bethune Hospital, Shanxi Academy of Medical Sciences, Third Hospital of Shanxi Medical University, Tongji Shanxi Hospital Taiyuan City China; ^2^ Third Hospital of Shanxi Medical University, Shanxi Bethune Hospital, Shanxi Academy of Medical Sciences, Tongji Shanxi Hospital Taiyuan China

**Keywords:** head and neck cancers, implantation metastasis of carcinoma, minimally invasive surgery, piecemeal resection of tumor, principles of oncologic surgery

## Abstract

Minimally invasive piecemeal resection for laryngeal neuroendocrine carcinoma may raise tracheotomy site implantation metastasis risk; adhere to oncologic surgical principles and strengthen postoperative surveillance to improve outcomes.

## Graphical abstract

We report a case of implantation metastasis of laryngeal neuroendocrine carcinoma at the tracheotomy site following minimally invasive piecemeal resection. This case highlights the risk of iatrogenic tumor seeding and emphasizes strict adherence to oncologic surgical principles, including en bloc resection, tumor‑free technique, and long‑term surveillance for improved patient outcomes.

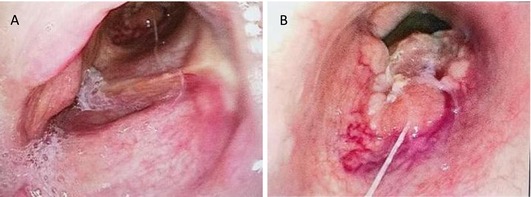

## 1 Introduction

Tumor implantation metastasis related to surgical procedures was identified by surgical pioneers a century ago, with William Stewart Halsted's 1894 radical mastectomy research laying the foundation for oncologic surgery [1]. Its core tenets—en bloc resection, clear surgical margins, regional lymph node dissection, and tumor‐free technique—have significantly reduced local tumor recurrence and improved patient survival rates. With the development of oncologic surgery, minimally invasive techniques have become increasingly important in the treatment of head and neck tumors [2, 3]. Yet routine piecemeal resection in such surgeries has been associated with an increased risk of implantation metastasis and local recurrence, a clinical challenge that requires attention [4].

Laryngeal neuroendocrine carcinoma (LNEC) is a rare subtype of neuroendocrine carcinoma (NEC) that mostly originates in the gastrointestinal tract and lower respiratory system [5]. LNEC is an aggressive malignant tumor with the potential for widespread metastasis, and most clinical cases are pathologically classified as moderately or poorly differentiated. For treatment, moderately/well‐differentiated LNEC is mainly managed with surgery supplemented by radiotherapy and/or chemotherapy, while poorly differentiated LNEC adopts a combined approach of radiotherapy/chemotherapy and surgery [6].

Iatrogenic implantation metastasis of head and neck malignancies has been reported in multiple surgical‐related sites, including reconstructive flap donor sites, laparoscopic port sites, and respiratory/digestive tract stoma sites such as tracheotomy or gastrostomy sites. Such metastasis typically occurs at traumatic sites associated with the primary tumor, often linked to surgical procedures or connected anatomical pathways that facilitate tumor cell translocation. This case report details a 67‐year‐old male patient with primary LNEC who developed implantation metastasis at the tracheotomy site after minimally invasive piecemeal resection, aiming to emphasize the importance of abiding by oncologic surgical principles in minimally invasive surgery and strengthening postoperative surveillance for this rare malignant tumor.

## 2 Case History/Examination

A 67‐year‐old male patient presented in July 2000 with a foreign body sensation in the pharynx. Laryngoscopic examination revealed a 1.5 cm smooth‐surfaced tumor on the laryngeal surface of the epiglottis, with no involvement of the anterior commissure or vocal cords. CT showed a soft‐tissue nodule at the root of the laryngeal surface of the epiglottis, with clear borders, a regular shape, and homogeneous internal density, measuring approximately 1.47 × 1.14 cm. During preoperative anesthesia, the patient unexpectedly developed laryngospasm, necessitating emergency tracheostomy for airway securement. The tumor was resected piecemeal using plasma technology under suspension laryngoscopy. We cauterized the base of the mass without resecting the epiglottis. Histopathological examination confirmed a well‐differentiated NEC. The patient declined adjuvant therapy and was followed closely.

In March 2022, follow‐up examination revealed a recurrent smooth tumor at the original site on the laryngeal aspect of the epiglottis, confirmed by biopsy as NEC. The mass measured 1.7 × 1.1 cm and no cervical lymph node metastasis or distant metastasis was detected. The patient subsequently underwent tracheotomy and open horizontal partial laryngectomy, with good recovery and continued follow‐up. The patient still did not receive adjuvant therapy.

## 3 Differential Diagnosis, Investigations and Treatment

**FIGURE 1:**
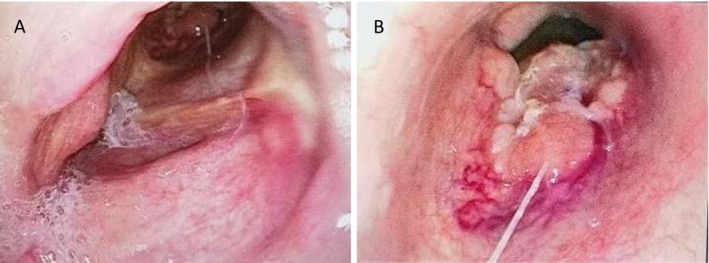
Laryngoscopy in September 2023. (A) Laryngoscopy demonstrating absent epiglottis, with smooth mucosa observed in the glottic and subglottic regions without any visible protrusions. (B) Laryngoscopy demonstrating irregular mass occupying nearly 80% of the anterior circumference of the tracheal circumference.

**FIGURE 2:**
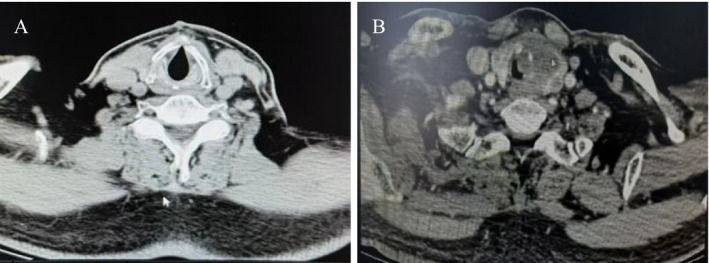
Neck CT in September 2023. (A) CT scan demonstrated no tumor detected in the glottic region. (B) CT demonstrating irregular mass occupying nearly 80% of the anterior circumference of the tracheal circumference.

In September 2023, the patient developed exertional dyspnea. Laryngoscopy showed no new lesions in the residual laryngeal cavity, but a cauliflower‐like neoplasm was observed in the anterior trachea at the previous tracheostomy site, occupying approximately 80% of the tracheal circumference (Figure 1). For the cauliflower‐like neoplasm found at the tracheotomy site, differential diagnosis was focused on excluding benign lesions and local tumor extension: Benign tracheal neoplasms (e.g., tracheal papilloma, inflammatory granuloma): excluded by pathological biopsy confirming NEC that was consistent with the primary tumor's pathological type. Local extension of the primary laryngeal tumor: ruled out by laryngoscopy and CT showing no new lesions in the residual laryngeal cavity, with normal tissue separating the tracheal mass from the original primary lesion site. Biopsy confirmed NEC. Neck contrast‐enhanced CT revealed an irregular mass (6 × 5 cm) at the level of the first to third tracheal rings with ill‐defined margins involving the left thyroid lobe and upper esophagus, as well as enlarged lymph nodes in the right neck levels III‐IV (Figure 2). Notably, no new lesions were found in the tongue base or residual larynx. Whole‐body PET‐CT showed no distant metastasis.

## 4 Conclusion and Results

The patient underwent a left partial laryngectomy, left thyroid lobectomy, partial tracheal resection, right neck levels II‐IV and bilateral level VI lymph node dissection, and tracheostomy. The tumor was completely resected, including partial laryngeal tissue, the left thyroid, and nearly the entire circumference of the first to fourth tracheal rings. The tracheal ring stumps were sutured to the skin with interrupted sutures to create a tracheostomy stoma. Frozen section pathology of the laryngeal and tracheal margins showed no carcinoma. Postoperative pathology confirmed a NEC, grade 2 (NET, G2), with metastatic carcinoma in 6 out of 32 right cervical lymph nodes. Adjuvant radiotherapy (60 Gy) began four weeks postoperatively. In April 2025, the patient exhibited significant weight loss, and PET‐CT revealed multiple lung metastases. The patient passed away half a month later.

## 5 Discussion

The adoption of minimally invasive techniques makes piecemeal resection a commonly employed surgical approach [7]. Reported iatrogenic implantation metastases in head and neck malignancies include: tumor metastasis at donor sites for reconstructive flaps (e.g., pectoralis major, anterolateral thigh, or temporalis muscle flaps) [8]; tumor metastasis at laparoscopic port sites (e.g., chest wall metastasis after endoscopic thyroidectomy) [9], and tumor metastasis along the respiratory or digestive tracts (e.g., tracheotomy or gastrostomy sites) [10]. These cases demonstrate that implantation metastasis primarily occurs at traumatic sites coexisting with the primary tumor, often related to surgical procedures or connected pathways, facilitating tumor cell translocation.

In this case, the recurrent tumor was located at the previous tracheotomy site, with normal tissue separating it from the primary lesion, suggesting implantation metastasis at the tracheotomy site. Since both prior surgeries involved tracheotomy, it is unclear whether the implantation occurred during the first or second procedure. However, the first surgery, which employed minimally invasive piecemeal resection, posed a higher risk of implantation metastasis. This case underscores the importance of achieving adequate surgical margins. The recurrence at the primary site after the first surgery was likely caused by insufficient margins. Thus, minimally invasive surgery for head and neck tumors should not abandon traditional oncologic surgical principles. The ultimate goal of radical resection remains complete tumor removal.

For eligible radical procedures, both en bloc and piecemeal resections can achieve complete tumor excision. However, given the higher risks of positive margins and implantation metastasis associated with piecemeal resection, traditional oncological surgery principles—such as prioritizing en bloc resection if possible, maintaining a tumor‐free technique, and ensuring clear margins—remain critically relevant in contemporary head and neck oncologic surgery.

## Author Contributions

**Rui Hou:** conceptualization, data curation, formal analysis, investigation, methodology, validation, visualization, writing – original draft. **Xiaolong Hao:** conceptualization, data curation, formal analysis, investigation, methodology, validation, visualization, writing – original draft. **Shuxin Wen:** supervision. **Yuhao Zhang:** methodology. **Tian Wang:** investigation. **Xiaolin Ji:** project administration. **Yueqin Wang:** data curation.

## Funding

This study was not supported by any funding agency or grant.

## Ethics Statement

The Ethics Committee of Shanxi Bethune Hospital has approved this project. Furthermore, we confirm that all applicable rules and procedures have been implemented in accordance with relevant guidelines, and informed consent forms have been obtained. All data were derived from all participants and were collected after the relevant clinical trials, in compliance with the principles of clinical practice and the Declaration of Helsinki.

## Consent

Written informed consent was obtained from the patient to publish this report in accordance with the journal's patient consent policy. We confirm that the manuscript has been read and approved by all named authors and that no other persons have satisfied the criteria for authorship but are not listed. We further confirm that all authors have approved the order of authors noted in the manuscript. We confirm that we have given that there are no objections to publication, including the timing of publication, concerning intellectual property.

## Conflicts of Interest

The authors declare no conflicts of interest.

## Data Availability

The data that support the findings of this study are available from the corresponding author upon reasonable request.
